# NIHSS Consciousness Score Combined with ASPECTS is a Favorable Predictor of Functional Outcome post Endovascular Recanalization in Stroke Patients

**DOI:** 10.14336/AD.2020.0709

**Published:** 2021-04-01

**Authors:** Zhe Cheng, Xiaokun Geng, Gary B Rajah, Jie Gao, Linlin Ma, Fenghai Li, Huishan Du, Yuchuan Ding

**Affiliations:** ^1^Department of Neurology and Stroke Center, Luhe Hospital, Capital Medical University, Beijing, China.; ^2^China-America Institute of Neuroscience, Luhe Hospital, Capital Medical University, Beijing, China.; ^3^Department of Neurosurgery, Wayne State University School of Medicine, Detroit, MI, USA.; ^4^Department of Neurosurgery, Jacobs School of Medicine and Biomedical Sciences, University at Buffalo, Buffalo, New York, USA.; ^5^Department of Neurosurgery, Gates Vascular Institute at Kaleida Health, Buffalo, New York, USA.; ^6^Department of Neurosurgery, Munson Medical Center, Traverse City, MI, USA.

**Keywords:** functional prognosis, large vessel occlusions, mechanical thrombectomy, Receiver Operating Characteristic curves, prediction method

## Abstract

Although revascularization rates after endovascular thrombectomy for large vessel acute ischemic stroke (AIS) are high (71%), only 46% of patients achieve functional independence at 90 days. The present study was designed to explore a new method for predicting the functional prognosis of AIS patients after endovascular recanalization. A total of 200 anterior circulation stroke patients who received endovascular therapy were enrolled. Logistic regression analysis of clinical characteristics on functional independence were performed. The predictive power of sub-items in National Institute of Health stroke scale (NIHSS) and the combination of NIHSS consciousness and Alberta Stroke Program Early CT Score (ASPECTS) on functional independence were assessed by Receiver Operating Characteristic (ROC) curves and the latter was compared with 3 previously published prediction models by AUC (the area under ROC curve). The AUC for the NIHSS consciousness score to predict functional independence was higher than whole NIHSS and other sub-items (0.716 v 0.705, 0.586, 0.573, 0.552 and 0.559). Low NIHSS consciousness score, high ASPECTS score, short time from onset to recanalization, and high rate of successful recanalization were demonstrated to be significantly associated with the functional independence (OR 0.697, 2.226, 0.994 and 28.643). The prediction power of the combination was significantly better than NIHSS and ASPECTS alone (AUC 0.793 v 0.705 and 0.752). Compared with 3 other prediction models, the combination was found to be the strongest predictor for functional independence (AUC 0.793 v 0.791, 0.671 and 0.564). NIHSS which has been shown to be a strong predictor of functional outcomes after endovascular recanalization is largely dependent on the consciousness component. NIHSS consciousness score combined with ASPECTS appears to be a favorable predictor of functional independence. These findings may have broad reaching effects for isolated centers around the world without advanced imaging for triage and prognostication.

Compared with intravenous rt-PA, endovascular therapy (EVT) leads to an overall higher degree of recanalization in acute ischemic stroke (AIS) patients with large vessel occlusions (LVO) and is associated with better short and long-term prognosis [1-3]. The recently published five multicenter prospective randomized trials have demonstrated the benefit of second-generation mechanical thrombectomy devices (primarily stent retrievers) among patients with AIS due to LVO [4-8], with a high rate of successful revascularization (77%)[9]. However, not all successfully recanalized patients progressed to pre morbid functionality after EVT with randomized trials on thrombectomy showing a 3-month mRS score (0-2) ranging from 32.6% (MR CLEAN) to 71.4% (Extend-IA) [1]. It is well-known that futile reperfusion can occur despite successful revascularization with failures to improve functional outcome [10].

To address the poorer-than-expected clinical outcomes despite successful EVT, there is a growing emphasis on improving patient selection using neuroimaging, a powerful predictor of EVT outcomes, to estimate the baseline core infarct size [11]. Despite the availability of advanced imaging systems, head computed tomographic (CT) remains the most commonly used imaging modality within 6 hours of stroke onset before EVT given its low cost, and availability throughout the world [11]. Alberta Stroke Program Early CT Score (ASPECTS) is a semi-quantitative grading system that measures the extent of early ischemic changes [12] and ASPECTS on baseline non contrast CT (NCCT ASPECTS) ≥6 is typically utilized to select appropriate patients prior to mechanical thrombectomy according to guidelines [1, 13-15]. Additionally, several prognostic factors were considered to be associated with functional outcome of AIS patients after vessel recanalization, such as National Institute of Health Stroke Scale (NIHSS), age, sex, occlusion site and factor risk [16-18].

The NIHSS is a 15-item scale that quantifies neurological deficits in several domains and it is commonly used in measuring stroke severity for patients with AIS [19]. Literature has indicated that NIHSS score was independently associated with functional prognosis of patients with AIS treated with or without EVT [10, 16, 20-24]. Additionally, NIHSS was considered to be a better predictor of the functional outcome after endovascular recanalization than other factors, such as hemineglect, female sex, atrial fibrillation, and no history of stroke or prestroke handicap [25]. According to 2019 AHA/ASA guidelines, NIHSS was recommended to assess stroke severity and for selecting groups of patients for EVT [15]. However, the key determinant of NIHSS to predict EVT outcomes remain unclear. Few studies were reported on the correlation between sub-items of NIHSS score and AIS. Among the NIHSS sub-items, motor, level of consciousness and ataxia scores in NIHSS were found associated with discharge disposition in patients with minor stroke [26]. In another study, severe facial palsy and visual field defect were shown to be associated with poor collateral status in acute stroke with middle cerebral artery (MCA) occlusion [27].

In this study, we aimed to screen for these prognostic factors and explore a new method which predicts functional prognosis of AIS patients within 6 hours from stroke onset after endovascular recanalization.

## MATERIALS AND METHODS

### Patients

We conducted a retrospective study with consecutive patients who underwent EVT for AIS with an LVO in anterior circulation from January 1, 2017 to October 1, 2019 in the Stroke Center, Beijing Luhe Hospital Capital Medical University. The indications for EVT were as follows: 1) Age ≥18, 2) Clinical diagnosis of AIS, 3) LVO in the anterior circulation demonstrated with predominately CT angiography (CTA), occasionally Digital subtraction angiography (DSA) [Thrombolysis in Cerebral Infarction (TICI) score=0], 4) NIHSS score 6-25 and an average ASPECTS score >5, 5) Less than 6 hours from stroke onset to groin puncture. Exclusion criteria were as follows:1) Persistent arterial blood pressure >185/110 mmHg, 2) Pregnancy, 3) Blood glucose <2.7 or >22.2 mmol/L, 4) Laboratory evidence of coagulopathy (platelet count <40×10^9^/L, APTT>50 seconds, or INR >3.0), 5) Cranial imaging revealing an infarction greater than one third of the MCA territory, 6) Pre-stroke modified Rankin Score (mRS)>2.

### Administration of rt-PA and bridging therapy

Patients who were eligible for intravenous thrombolysis received recombinant tissue plasminogen activator (rt-PA) at a dose of 0.9 mg/kg (10% of the dose was given as a bolus within 1 min followed by a 60 min infusion) while endovascular intervention was simultaneously being prepared. All endovascular procedures were performed under local anesthesia with/without sedative agents. At the same time with rt-PA infusion, patients were transferred to the angiography suite for DSA. If LVO was confirmed by DSA, the bridging EVT would be conducted. Interventional strategies were left to the discretion of the treating interventionalist, including the choice of stent retrievers, aspiration, stenting or other necessary devices. Combination of stent retriever and intracranial support catheter was used for mechanical thrombectomy most frequently.

### Imaging Evaluation

A brain CT scan was obtained at baseline and immediately after endovascular intervention to assess ischemic stroke burden and intracerebral cerebral hemorrhage (ICH). The vast majority of patients (96%) patients had received CTA at admission to confirm LVO. Magnetic resonance imaging (MRI) was routinely performed at 24 hours after vessel recanalization. If unable to complete MRI or any sign of neurological deterioration occurred within 24 hours, another head CT scan would be performed. A head and neck CTA or magnetic resonance angiography (MRA) was performed within one week. Any ICH at 7 days or discharge was recorded and classified into 5 categories according to the European Cooperative Acute Stroke Study II (ECASS 2) [28]. Symptomatic ICH (sICH) was defined as radiographic ICH with a corresponding increase of 4 or greater on the NIHSS. ASPECTS scoring depended on the baseline CT before EVT. Target vessel recanalization was assessed by TICI scale [29]. Successful vessel recanalization was defined as TICI≥2b. ASPECTS and TICI scoring were assessed by 2 neuroradiologists.

### Neurological Status Assessments

NIHSS was assessed depending on examination of the nervous system in an emergency room. Functional outcome was assessed by mRS score and good outcome (functional independence) was defined as mRS score of 0 to 2 at 90 days after stroke onset. The mRS score was obtained by personnel who were blinded to admission NIHSS score. Both face-to-face in the consultation room and telephone interviews were used in the study to calculate 90-days mRS score. Death, cause of death, and any other systemic bleeding complications were recorded during 3 months after stroke onset. The procedural metrics of EVT were also recorded including time from onset to recanalization (TOR), time from treatment to recanalization (TTR), number of passes of the retriever stent (NOP). Consciousness Score in NIHSS (0-7) and ASPECTS (0-10) were combined for predicting functional independence of AIS patients after endovascular recanalization. Depending on lower consciousness scores in NIHSS and higher ASPECTS scores associated with better functional prognosis, the computational formula of the combination was designed as 7- consciousness scores in NIHSS+ ASPECTS scores. On the same cohort data in this study, the prediction power of the combination was compared with 3 previously published prediction models by Receiver operating characteristic (ROC) curves, including Pittsburgh Response to Endovascular Therapy (PRE) score [30], Stroke Prognostication Using Age and National Institutes of Health Stroke Scale (SPAN) index [23], and Totaled Health Risks in Vascular Events (THRIVE) score [31].

### Statistical Analysis

The Kolmogorov-Smirnov test for normality and the equal variance test were performed before any statistical analysis was used. For continuous data, 2-sided *t* test for independent samples or Mann-Whitney U test was performed to detect differences between groups. For binary data, χ2 or Fisher exact tests were performed when appropriate between groups. Logistic regression analysis was performed with functional outcomes. ROC curves were performed to assess efficiency of sub-items in NIHSS and combination of NIHSS consciousness and ASPECT score on prediction functional independence in the study. The area under ROC curve (AUC) is a frequently used tool for assessing the overall accuracy of a diagnostic marker (scoring range 0.5-1, higher core indicating better efficacy)[32].The significance level was set at *p*<0.05. All *p*<0.05 were two-sided. Statistical analysis was performed using SPSS version 23.

**Table 1:** Demographic and clinical characteristics of all patients.

	All Patients (n=200)
Age, mean (SD), y	66.2 ± 10.8
Sex ratio (male/female)	116/84
NIHSS at onset, mean (SD), point	16.7 ± 3.6
ASPECTS, median (SD), point	8.2 ± 1.3
Risk factors, n (%)	
Hypertension	137 (68.5)
Diabetes mellitus	41 (20.5)
Atrial fibrillation	83 (41.5)
Smoking	60 (30.0)
Occlusion site, n (%)	
M1	138 (69.0)
D-ICA	39 (19.5)
P-ICA	23 (11.5)
IV rt-PA, n (%)	83 (41.5)
NOP, median (SD), time	1.5 ± 0.8
TTR, mean (SD), min	64.0 ± 29.8
TOR, mean (SD), min	246.5 ± 81.1
TICI≥2b, n (%)	162 (81.0)
mRS 0-2 at 3 months, n (%)	112 (56)
Symptomatic ICH, n (%)	11 (5.5)

Abbreviations: NIHSS, the National Institute of Health Scale Score; ASPECTS, Alberta Stroke Program Early Computed Tomography Score; mRS, modified Rankin score; M1, middle cerebral artery segment; D-ICA, distal ICA; P-ICA, proximal segment of internal carotid artery; IV rt-PA, intravenous recombinant tissue plasminogen activator; TOR, time from onset to recanalization TTR, time from treatment to recanalization; NOP, number of passes of retriever stent; TICI, thrombolysis in cerebral infarction.

## RESULTS

### Demographic and clinical characteristics

A total of 200 anterior circulation stroke patients who received EVT were enrolled in the current study. Baseline demographics and characteristics of the 200 patients are summarized in Table 1. The mean age was 66.2 ± 10.8 years and mean NIHSS score at admission was 16.7 ± 3.6. A total of 138 (69.0%) patients had an occlusion in MCA, 39 (19.5%) in distal ICA (internal carotid artery) and 23 (11.5%) in proximal segment of ICA. The mean ASPECTS score in patients with anterior circulation occlusion was 8.2 ± 1.3. A total of 83 patients (41.5%) were treated with IV rt-PA prior to EVT. The average time from stroke onset to recanalization (TOR) was 246.5 ± 81.1min, and from initiation of treatment to recanalization (TTR) was 64.0 ± 29.8 min. The average number of passes of the stent retriever was 1.5 ± 0.8. One hundred sixty-two subjects (81.0%) achieved TICI≥2b and 112 (56%) achieved mRS 0-2 at 90 days after stroke onset.

**Table 2:** Analysis of demographic and clinical characteristics between good and poor outcome groups.

	Good outcome (n=112)	Poor outcome (n=88)	*P* Value
Age, median (SD), y	63.9 ±10.2	69.2 ± 11.0	.000^*^
Male, n (%)	78 (69.6)	38 (43.2)	.000^*^
NIHSS at onset, median (SD), point	15.6 ± 3.1	18.2 ± 3.7	.000^*^
ASPECTS, median (SD), point	8.8 ± 0.9	7.5 ±1.3	.000^*^
Risk factors, n (%)			
Hypertension	77 (68.8)	60 (68.2)	1.000
Diabetes mellitus	17 (15.2)	24 (27.3)	.051
Atrial fibrillation	44 (39.3)	39 (30.8)	.563
Smoking	36 (32.1)	19 (21.6)	.112
Occlusion site, n (%)			
M1	79 (70.5)	59 (67.0)	.645
D-ICA	15 (13.4)	24 (27.3)	.019^*^
P-ICA	18 (16.1)	5 (5.7)	.026^*^
IV rt-PA, n (%)	46 (41.1)	37 (42.0)	1.000
NOP, median (SD), time	1.4 ± 0.69	1.7 ± 0.85	.004^*^
TTR, median (SD), min	59.9 ± 28.0	70.1 ± 31.5	.002^*^
TOR, median (SD), min	235.6 ± 80.2	261.4 ± 81.0	.017^*^
TICI ≥2b, n (%)	108 (96.4)	54 (61.4)	.000^*^
sICH, n (%)	3 (2.7)	8 (9.1)	.096

Abbreviations: NIHSS, the National Institute of Health Scale Score; ASPECTS, Alberta Stroke Program Early Computed Tomography Score; mRS, modified Rankin score; M1, middle cerebral artery segment; D-ICA, distal ICA; P-ICA, proximal segment of internal carotid artery; IV rt-PA, intravenous recombinant tissue plasminogen activator; NOP, the number of passage of retriever stent; TOR, time from onset to recanalization TTR, time from treatment to recanalization; TICI, thrombolysis in cerebral infarction; sICH, symptomatic intracranial hemorrhage;

* *p*< 0.05.

### Comparison between good and poor outcome groups

Among all subjects enrolled in the study, 112 (56%) patients achieved good outcome (mRS 0-2) at 90 days after stroke onset. When comparing patient demographics to poor outcomes groups, the patients were younger in good outcome group (63.9 vs 69.2, *p*<0.001) with a greater proportion of men than women (69.6 vs 43.2%, *p*<0.001). Not surprisingly, a significantly lower baseline NIHSS (15.6 vs 18.2, *p*<0.001) and higher ASPECTS score (8.8 vs 7.5, *p*<0.001) were found in good outcome group. Additionally, significantly fewer patients in the good outcome group suffered from occlusion in distal ICA (terminus) (13.4 vs 27.3%, *p*=0.019) and significantly more patients suffered from occlusion in proximal segment of ICA (16.1 vs 5.7%, *p*=0.026) in the good vs poor outcome group. Moreover, patients in good outcome group had a significantly shorter TTR (59.9 vs 70.1, *p*=0.002) and TOR (235.6 vs 261.4, *p*=0.017). A significantly lower NOP was noted in the good outcome group (1.4 vs 1.7 *p*=0.004). The rate of successful recanalization (TICI≥2b) was significantly higher in good outcome group versus the poor outcome group (96.4 vs 61.4%, *p*<0.001). There were no significant differences in vascular risk factors, IV rt-PA and sICH between two groups (Table 2).

### Comparison between sub-items of NIHSS on prognosis

In order to assess the effect of NIHSS on predicting functional independence, sub-items of NIHSS were analyzed between good and poor groups (Table 3). In the good outcome group, lower scores on consciousness and language were found at admission as compared to the poor outcome group, the difference was significant (1.9 vs 3.5, *p*<0.000; 1.8 vs 2.1, *p*=0.045). There was no significant difference in scores for gaze, facial palsy and motor between two groups (*p*>0.05). Due to reduction of consciousness in most of the patients after stroke onset, scoring of visual, limb ataxia, sensory and dysarthria were difficult and were not assessed.

**Figure 1.**
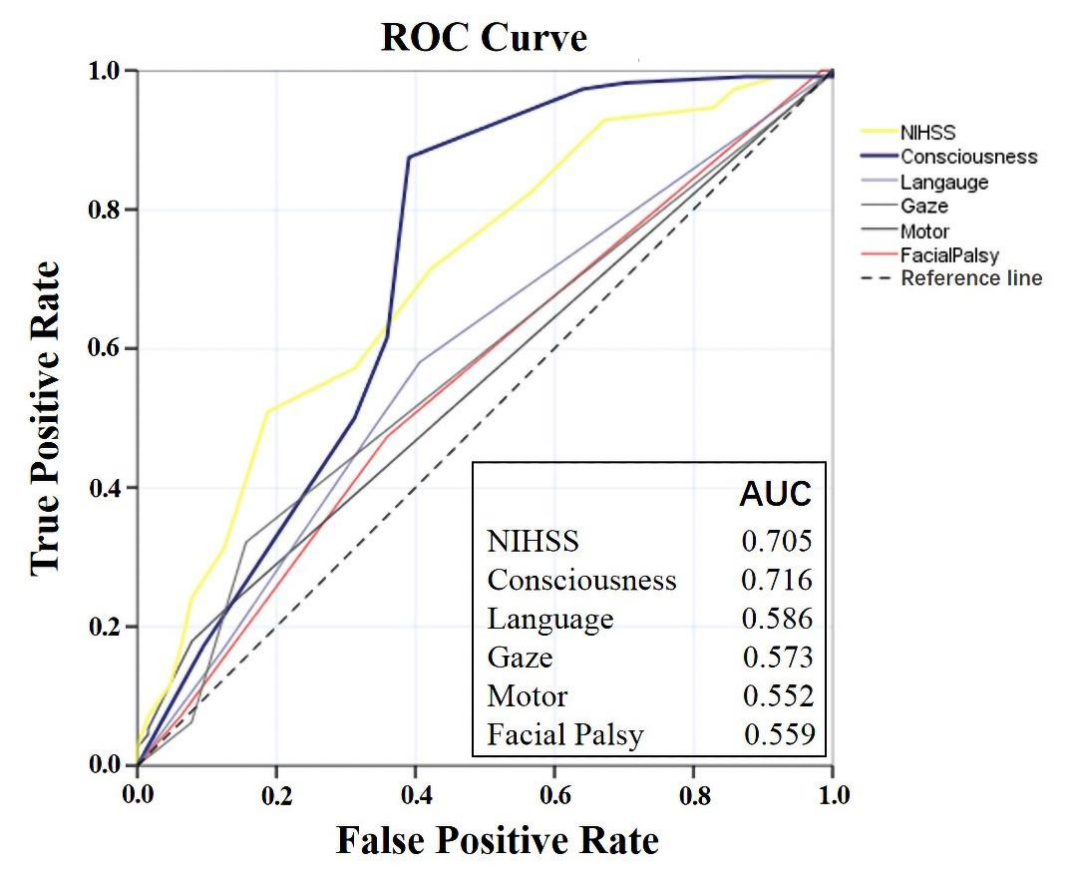
Comparison of predictive power among the NIHSS and each sub-item score on functional independence after endovascular recanalization. The AUC is provided, suggesting that consciousness score (0.716) in NIHSS was better able to predict functional outcome than all others. ROC, receiver operating characteristic; AUC, area under ROC curve, a frequently used tool for assessing the overall accuracy of a diagnostic marker (scoring range 0.5-1, higher core indicating better efficacy); NIHSS, the National Institute of Health Scale Score.

The efficacy of all sub-item score in NIHSS to predict functional independence after endovascular recanalization was assessed by ROC curve. The prediction power of consciousness score in NIHSS (AUC=0.716, 95% CI 0.629 to 0.804) had a better fit than other sub-items, including Language (AUC=0.586, 95% CI 0.498 to 0.673, *p*<0.001), Gaze (AUC=0.573, 95% CI 0.486 to 0.660, *p*<0.001), Motor (AUC=0.552, 95% CI 0.466 to 0.639, *p*<0.001), Facial palsy (AUC=0.559, 95% CI 0.471 to 0.647, *p*<0.001). The prediction power of consciousness score in NIHSS was not statistically superior to the whole NIHSS (AUC=0.705, 95% CI 0.624 to 0.785, *p*=0.18) although a trend was observed (Fig. 1). Patients without successful vessel recanalization were not analyzed in this analysis.

**Table 3:** Analysis of sub-items of NIHSS on functional independence between good and poor outcome groups.

	Good outcome (n=112)	Poor outcome (n=88)	*P* value
Consciousness	1.9 ± 1.8	3.5 ± 2.3	.000*
Gaze	1.6 ± 0.6	1.8 ± 0.6	.108
Visual	-	-	-
Facial Palsy	1.5 ± 0.6	1.6 ± 0.6	.119
Limb Ataxia	-	-	-
Sensory	-	-	-
Language	1.8 ± 1.2	2.1 ± 1.1	.045^*^
Dysarthria	-	-	-
Extinction and Inattention	-	-	-

* *p*< 0.05

**Table 4:** Logistic regression analysis of the demographic and clinical characteristics on functional independence.

	Odds ratio	95% CI	*P* Value
consciousness score in NIHSS	0.697	0.554-0.887	.002*
ASPECTS	2.226	1.557-3.184	.000*
TOR	0.994	0.988-0.999	.025*
TICI ≥2b	28.643	5.846-140.337	.000*

Abbreviations: Age, sex, language score in NIHSS, consciousness score in NIHSS, ASPECTS score, occlusion site, NOP, TOR, TTR and rate of TICI ≥2b were included in the regression analysis. NIHSS, the National Institute of Health Scale Score; ASPECTS, Alberta Stroke Program Early Computed Tomography Score; D-ICA, distal ICA ; P-ICA, proximal segment of internal carotid artery; NOP, number of passes of retriever stent; TOR, time from onset to recanalization; TTR, time from treatment to recanalization; * *p*< 0.05.

### Logistic regression analysis on functional prognosis

Logistic regression analysis of the consciousness in NIHSS, demographics and other clinical characteristics on functional prognosis at 90 days were summarized in Table 4. Only 4 assessed factors, including low NIHSS of consciousness, high rate of successful recanalization, high ASPECTS score, and short TOR were correlated with the functional independence at 90 days (OR 0.697, 95% CI 0.554-0.887, *p*<0.05; OR 28.643, 95% CI 5.846-140.337, *p*<0.001; OR 2.226, 95% CI 1.557-3.184, *p*<0.001; OR 0.994, 95% CI, 0.988-0.999, *p*<0.05). However, other key factors, such as age, sex, language score in NIHSS, occlusion site, NOP and TTR revealed no correlation with the functional independence at 90 days.

**Figure 2.**
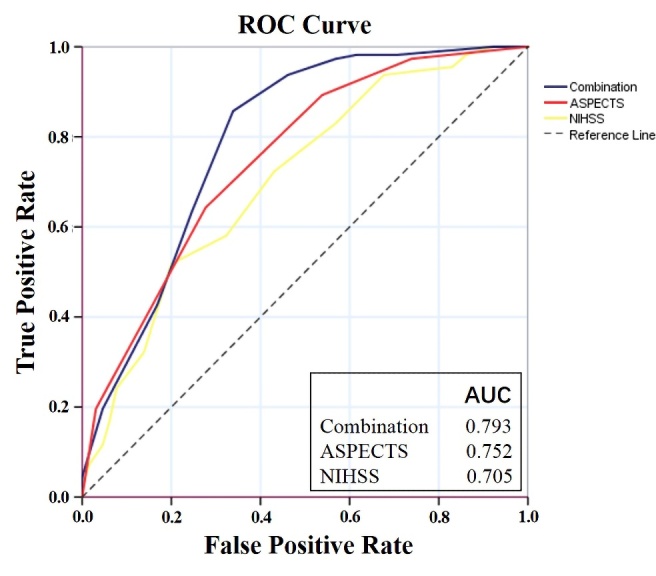
Comparison of predictive power among NIHSS, ASPECTS and the combination on the functional independence after endovascular recanalization. The AUC suggests that the combination (0.793) was better able to predict functional outcome than NIHSS and ASPECTS each alone. ROC, receiver operating characteristic; AUC, area under ROC curve, a frequently used tool for assessing the overall accuracy of a diagnostic marker (scoring range 0.5-1, higher core indicating better efficacy); NIHSS, the National Institute of Health Scale Score; ASPECTS, Alberta Stroke Program Early Computed Tomography Score.

**Figure 3.**
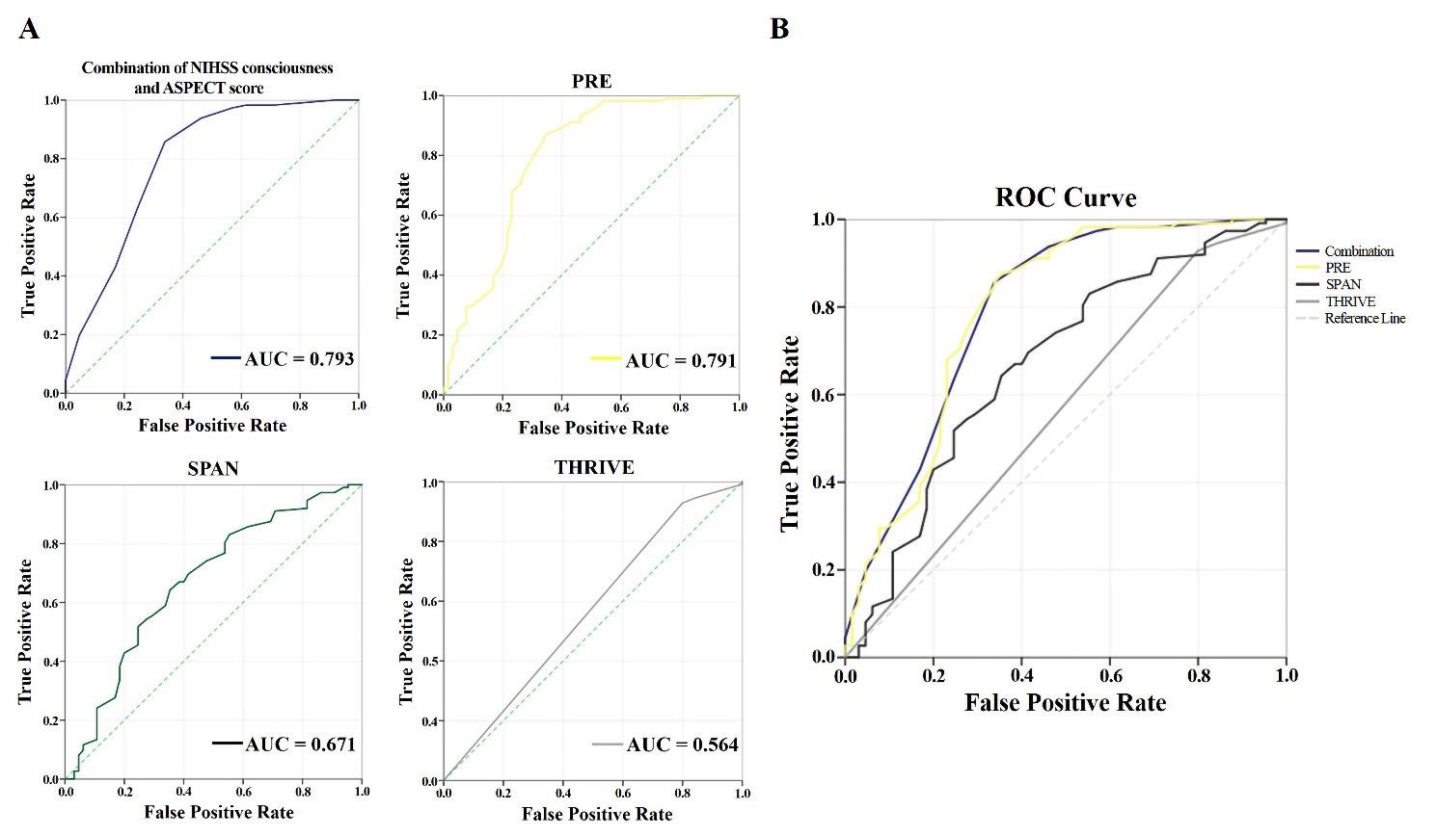
Comparison of predictive power among the combination of NIHSS consciousness and ASPECTS score, PRE, SPAN and THRIVE on the functional independence after endovascular recanalization. (A) Comparison of predictive power between the combination of NIHSS consciousness and ASPECTS score and other scoring models on functional independence after endovascular recanalization by AUC was given. (B) The figure suggesting combination of NIHSS consciousness and ASPECTS score was the strongest predictor for functional outcome as compared to other 3 models (AUC 0.793 v 0.791, 0.671 and 0.564). ROC, receiver operating characteristic; AUC, area under ROC curve, a frequently used tool for assessing the overall accuracy of a diagnostic marker (scoring range 0.5-1, higher core indicating better efficacy); PRE, Pittsburgh Response to Endovascular Therapy score; SPAN, Stroke Prognostication Using Age and NIHSS index; and THRIVE, Totaled Health Risks in Vascular Events score.

### Further analytics of functional prognosis depending on NIHSS consciousness and ASPECTS score

Rate of functional independence was compared depending on the degree of disturbance of consciousness at admission (consciousness score 0-3 and 4-7 are defined as mild and severe disturbance of consciousness, respectively) and ASPECTS score (high ASPECTS score, 9-10 point; low ASPECTS score, 6-8 point) (Table 5). A significant higher rate of functional independence was found in mild disturbance of consciousness group as compared to patients with severe disturbance of consciousness (80.8% vs 26.2%, *p*<0.001). The similar rate of functional independence was found in patients with high ASPECTS score, significantly higher than patients with low ASPECTS score (85.4% vs 43.8%, *p*<0.001). Patients without successful vessel recanalization were not analyzed in the analysis.

### Prediction power of the combination of NIHSS consciousness and ASPECTS

The efficacy of the combination of NIHSS consciousness and ASPECTS score to predict functional independence was assessed by ROC curve. The AUC of the combination was 0.793 (95% CI 0.719 to 0.867) and optimal prediction threshold was 12.5 (sensitivity 0.857, specificity 0.662) for predicting functional independence. The prediction power of the combination of NIHSS consciousness score and ASPECTS was significantly better than either classic stroke scale in singularity [NIHSS AUC=0.705, 95% CI 0.624 to 0.785, *p*<0.001) or ASPECTS (AUC=0.752, 95% CI 0.677 to 0.827, *p*<0.05), respectively] (Fig. 2). On the same cohort data in this study, prediction power of the combination was better than SPAN (AUC=0.671, 95% CI 0.587 to 0.756, *p*<0.001) and THRIVE (AUC=0.564, 95% CI 0.474 to 0.654, *p*<0.001). Prediction power of the combination was not statistically superior to PRE (AUC=0.791, 95% CI 0.716 to 0.867, *p*=0.24) although a trend was observed (Fig. 3).

**Table 5:** Comparing functional independence between patients with mild and severe disturbance of consciousness or high and low ASPECTS score.

	Mild disturbance of consciousness (n=120)	Severe disturbance of consciousness (n=42)	*P* Value	High ASPECTS (n=89)	Low ASPECTS (n=73)	*P* Value
mRS (0-2) at 90 days, n (%)	97 (80.8)	11 (26.2)	0.000	76 (85.4)	32 (43.8)	0.000

Abbreviations: ASPECTS, Alberta Stroke Program Early Computed Tomography Score; mRS, modified Rankin score; TICI ≥2b, successful vessel recanalization; Consciousness score 0-3 and 4-7 are defined as mild and severe disturbance of consciousness respectively; 9,10 and 6-8 are defined as high and low ASPECTS score respectively; * *p*< 0.05.

## DISCUSSION

This study revealed 4 main periprocedural factors, including NIHSS, ASPECTS score, TOR and TICI score which affected the prognosis of AIS patients with LVO after EVT. Moreover, the correlation of AIS prognosis and NIHSS mostly depended on the score of consciousness but not on other sub-items. NIHSS consciousness score combined with ASPECTS offered a powerful prediction effect for functional independence after endovascular recanalization.

Previous studies have identified that women have worse functional outcomes after stroke without EVT[33, 34]. After receiving EVT in this study, female patients received lower rates of functional independence than men (43.2 vs. 69.6 %), suggesting sex may be a predictor of functional outcome. Younger patients were found to be more likely to achieve good outcome after EVT in this study [35]. Additionally, ICA occlusions have been previously considered as predictors of poor outcomes in AIS after EVT [16] and the reason may be related to increased difficulty recanalizing ICA occlusions, resulting in a longer time to recanalization [16]. However, in our study, when distinguishing distal and proximal segment of ICA occlusions, we found more patients with proximal segment of ICA occlusion and fewer patients with distal ICA occlusions achieved functional independence. The reason for this difference may be most of proximal segment of ICA occlusion were related to chronic stenosis possibly with a rich collateral network [36]. Consistent with previous studies, high ASPECTS score was demonstrated to be associated with functional independence of AIS patients after EVT in this study [13, 14].

High NIHSS score on admission has previously been correlated with poor outcomes after EVT by several studies[1], which was consistent with our findings. When the cohort was further analyzed depending on sub-items of NIHSS, the significant difference was found on consciousness and language score between patients with good and poor functional outcome. When we further analyzed the effect of sub-items of NIHSS for predicting functional prognosis by AUC, consciousness score in NIHSS revealed a better predictive effect than other sub-items in NIHSS (AUC 0.586 in Language, 0.573 in Gaze, 0.552 in Motor, 0.559 in Facial Palsy) on functional independence and a trend to precede NIHSS itself (AUC 0.716 vs. 0.705). Thus, we consider that the predictive power of NIHSS was largely dependent on the consciousness score, and other sub-items in NIHSS may even interfere with predictive efficacy. The different predictive power of sub-items in NIHSS on functional outcome after EVT, to our knowledge, has not been previously stated. Although the mechanism of poor consciousness being related to poor outcomes is not completely known, it is very likely that more severe disturbance of consciousness indicated a higher ischemic burden in most instances or critical parenchymal areas, leading to poor prognosis. Accordingly, the consciousness status should be carefully evaluated when patients are selected for EVT.

Logistic regression in the study revealed 4 factors were associated with functional prognosis. Only consciousness score in NIHSS and ASPECTS were the preoperative factors, thus assessing of those two factors may be more effective for selecting patients prior to EVT. Additionally, higher rates of functional independence were found in the mild disturbance of consciousness and high ASPECTS score group as compared to severe disturbance of consciousness and low ASPECTS score group (80.8% and 85.4% vs. 26.2% and 43.8%), respectively. The prediction power of the combination of these two factors (AUC=0.793) had a better fit than both NIHSS (AUC=0.705) and ASPECTS (AUC=0.752) used alone. Moreover, the combination depicts the best effect on predicting functional independence when compared with 3 previously reported prediction models (AUC=0.791 in PRE, 0.671in SPAN, 0.564 in THRIVE). The combined scoring process is concise and may be used by triage physicians quickly for preoperative assessment of patients with LVO before EVT. This would be especially critical in areas where resources are limited including access to advanced imaging, and low capacity facilities serving vast communities. Thus, the combination of NIHSS consciousness and ASPECTS score may be a favorable prediction model to help select patients for EVT and counsel families regarding possible outcomes. The score greater than 12.5 (sensitivity 0.857, specificity 0.662) may be more likely to achieve functional independence after endovascular recanalization in AIS.

In conclusion, NIHSS which has been shown to be a strong predictor of functional outcomes after endovascular recanalization is largely dependent on the consciousness component. NIHSS consciousness score combined with and ASPECTS appears to be a powerful predictor of functional independence, as compared to each alone. Further, the prediction power of the combination was better than the other two well-known predicting systems, SPAN and THRIVE. Therefore, the present results may have broad reaching effects for isolated centers around the world without advanced imaging for triage and prognostication.

## Limitation and future study

First, several uncollected data items may affect the primary endpoint in this study, for example, collateral status, pre-stroke mRS, type of device used for MT procedure, pressure and glycemic values were not collected; Second, the present study was hypothesis-generating, it had a retrospective design to screen the most relevant factors affecting functional prognosis after endovascular recanalization. A prospective study on combined NIHSS consciousness and ASPECTS is needed to confirm the result; Third, a validation cohort to assess and improve the efficiency of the combination model will be performed; Last, the effect of the combination model in other AIS patients should be assessed in the future.
